# Hepatocyte Expression of the Senescence Marker p21 Is Linked to Fibrosis and an Adverse Liver-Related Outcome in Alcohol-Related Liver Disease

**DOI:** 10.1371/journal.pone.0072904

**Published:** 2013-09-23

**Authors:** Aloysious Aravinthan, Giada Pietrosi, Matthew Hoare, James Jupp, Aileen Marshall, Clare Verrill, Susan Davies, Adrian Bateman, Nick Sheron, Michael Allison, Graeme J. M. Alexander

**Affiliations:** 1 Department of Medicine, University of Cambridge, Cambridge, United Kingdom; 2 Cancer Research UK Cambridge Institute, University of Cambridge, Cambridge, United Kingdom; 3 Department of Hepatology, University Hospital Southampton, Southampton, United Kingdom; 4 Department of Histopathology, Cambridge University Hospital, Cambridge, United Kingdom; 5 Department of Histopathology, University Hospital Southampton, Southampton, United Kingdom; Bambino Gesu' Children Hospital, Italy

## Abstract

**Background and Aim:**

Alcohol-related liver disease (ALD) remains a leading cause of liver-related morbidity and mortality. Age, fibrosis stage, MELD score and continued alcohol consumption predict outcome in everyday clinical practice. In previous studies increased hepatocyte nuclear area and hepatocyte expression of p21, both markers of senescence, were associated with increased fibrosis stage and a poor outcome in non-alcohol-related fatty liver disease, while increased hepatocyte nuclear area was related to liver dysfunction in ALD cirrhosis. This study, therefore, investigated the pattern of hepatocyte cell cycle phase distribution and hepatocyte p21 expression in relation to outcome in ALD.

**Methods:**

Liver sections from two cohorts were studied. The first comprised 42 patients across the full spectrum of ALD. The second cohort comprised 77 patients with ALD cirrhosis. Immunohistochemistry assessed hepatocyte expression of cell cycle phase markers and p21. Regenerating liver (n=12) and “normal” liver sections (n=5) served as positive and negative controls, respectively.

**Results:**

In the first cohort there was little cell cycle progression beyond G_1_/S phase and increased hepatocyte p21 expression (p<0.0001), which correlated independently with fibrosis stage (p=0.005) and an adverse liver-related outcome (p=0.03). In the second cohort, both hepatocyte p21 expression (p<0.001) and MELD score (p=0.006) were associated independently with an adverse liver-related outcome; this association was stronger with hepatocyte p21 expression (AUROC 0.74; p=0.0002) than with MELD score (AUROC 0.59; p=0.13). Further, hepatocyte p21 expression co-localised with increased hepatic stellate cell activation.

**Conclusions:**

The findings are consistent with impaired cell cycle progression beyond the G_1_/S phase in ALD. The striking independent associations between increased hepatocyte p21 expression and both fibrosis stage and an adverse liver-related outcome in both cohorts suggests hepatocyte senescence plays an important role in ALD. Measuring hepatocyte p21 expression is simple and cheap and in this series was a useful measure of long-term prognosis in ALD.

## Introduction

Alcohol-related liver disease (ALD) encompasses a broad spectrum of liver injury ranging from simple steatosis through alcohol-related hepatitis to cirrhosis and hepatocellular carcinoma [1–3]. The pathophysiology of ALD is complex, but hepatocyte oxidative stress is considered a key player, as alcohol consumption increases production of reactive oxygen species and lowers cellular antioxidant levels through a number of mechanisms [3,4]. Gender, genetics, the dose, duration and type of alcohol consumed influence the development of ALD [1–3,5]. Once established, age, fibrosis stage, severity scores such as the Model for End-Stage Liver Disease (MELD) and continued alcohol consumption after diagnosis (exceeding 10g/day) are useful indicators of prognosis [3,6].

The validity of clinical criteria in predicting outcome in ALD has been established, but less is known of liver histology in relation to prognosis. In one study increased hepatocyte nuclear area was related to hepatic dysfunction (prolonged prothrombin time and increased serum bilirubin) and hepatic decompensation (jaundice, ascites and encephalopathy) [7]. Increased hepatocyte nuclear area suggests hepatocyte senescence, since nuclear enlargement is a recognised morphological characteristic of cellular senescence [8,9]. However, it is unlikely that measuring hepatocyte nuclear area could be introduced readily into routine clinical practice as it is time consuming and not easily automated.

Irreversible cell cycle arrest that limits the proliferative potential of cells is a feature of cellular senescence and is mediated by p21, a universal cell cycle inhibitor [10]. p21 also contributes to the stability of cell cycle arrest long after the induction of senescence [10]. Thus, it plays a vital role in induction and maintenance of cellular senescence. Recent studies have demonstrated the potential utility of p21 expression as a prognostic marker in malignant disease [11]. However, less is known of the prognostic implications of p21 expression in chronic inflammatory conditions such as ALD.

A recent study demonstrated that markers of senescence including hepatocyte p21 expression predicted an adverse liver-related outcome in patients with non-alcohol related fatty liver disease [12]. This prompted the current study in which the aims were to determine the pattern of cell cycle phase distribution in hepatocytes in patients with ALD and to assess the association between hepatocyte p21 expression and clinical outcome.

## Materials and Methods

### Patients

Two cohorts of patients with ALD were studied. All patients gave a history of sustained excessive alcohol consumption (men >30g/d; women >20g/d). Other recognised causes of liver disease including chronic viral hepatitis, autoimmune liver disease, hereditary hemochromatosis, α1-antitrypsin deficiency and Wilson’s disease were excluded with appropriate investigations. All patients had routine haematology and biochemistry blood tests performed at the time of liver biopsy and were reviewed at least every 6 months until death, an adverse liver-related outcome or the censor point. Only those patients with complete follow-up data were included.

The first cohort comprised 42 patients within the full spectrum of ALD who underwent liver biopsy and remained under long-term follow-up at Cambridge University Hospitals. A second cohort comprised 77 patients with biopsy confirmed alcohol-related cirrhosis under long-term follow-up at University Hospital, Southampton, which was studied to confirm the findings in relation to outcome in the first cohort.

A composite primary endpoint was used for the analysis of an adverse liver-related outcome. An adverse liver-related outcome event was defined as liver-related death, liver transplantation or the development of hepatocellular carcinoma. Survival was determined as the time from liver biopsy; survivors were censored at last clinic appointment.

### Controls

Liver biopsy specimens were obtained from 12 patients during the regenerative phase of acute ischaemic-reperfusion injury following liver transplantation to serve as positive controls for expression of the full range of cell cycle phase markers. All of these patients made a rapid recovery after transplantation with normal liver function at discharge from hospital. All the liver biopsy specimens showed evidence of regeneration on H&E-stained sections.

Liver biopsy specimens were also obtained from background liver from five patients with colorectal cancer metastases, which served as negative controls for cell cycle phase markers. None of these patients had received chemotherapy prior to liver biopsy. Needle biopsies were distant to metastases with normal histology on H&E-stained sections.

### Liver biopsy specimens

All liver tissues were obtained from formalin-fixed paraffin-embedded liver needle biopsy specimens, in accordance with Cambridge University Hospitals and University Hospital, Southampton local research ethics committee guidelines and approval. All participants provided informed, written consent for the liver tissue to be used for research purposes. Patients were only included in the study if the biopsy exceeded 1.5cm in length.

### Immunohistochemistry

A Bond™ machine (Leica Microsystems), was used to perform automated immunohistochemistry. Unconjugated mouse monoclonal anti-Mcm-2 served as a marker of cell cycle entry (Novocastra; concentration 1:25). Unconjugated mouse monoclonal anti-Cyclin A (Novocastra; concentration 1:25) and unconjugated mouse monoclonal anti-PH3 (Upstate Biotechnology; concentration 1:500) served as markers of S phase and M phase respectively. Unconjugated mouse monoclonal anti-p21 served as a marker of cell cycle arrest (Dako; concentration 1:100).

Hepatocytes with brown-stained nuclei were considered positive for Mcm-2, cyclin A, PH3 and p21 whereas hepatocytes with blue-stained nuclei were considered negative; differences in the intensity of staining were not taken into consideration. Immunohistochemistry was assessed quantitatively to ensure objectivity. All images obtained from DotSlide digital microscope were analysed twice - using ScanR analysis software and manually. Photomicrographs of at least 20 lobular fields were analysed for each section (at least 1000 hepatocyte nuclei were counted). There was close correlation between the semi-automatic ScanR analysis and manual counting (r^2^=0.93; p<0.0001). Mcm-2 and p21 positive hepatocytes were expressed as a percentage of the total number of hepatocytes; Cyclin A and PH3 were expressed as percentage of the number of hepatocytes expressing Mcm-2.

Ten further sections, all from patients with ALD cirrhosis, were stained with unconjugated mouse monoclonal anti-Actin α-Smooth Muscle antibody (α-SMA; Sigma; concentration 1:2000), a marker of activated hepatic stellate cells. Qualitative assessment examined the geographic association between hepatic stellate cell activation and hepatocyte p21 expression.

### Interpretation of slides

Liver samples were assessed and scored using a modified Kleiner scoring system by specialist liver histopathologists (SD & AB), blinded to the immunohistochemistry results and clinical outcome. Architectural staining was performed using Gordon & Sweets’ reticulin and Chromotrope Aniline Blue (CAB) for assessment of fibrosis stage. Hematoxylin and Eosin (H&E) staining was used for quantification of steatosis grade and inflammation. Fibrosis was scored from 0 (none) to 4 (cirrhosis) and steatosis scored 0 (<5% parenchymal involvement under low- to medium-power evaluation) to 3 (>60% parenchymal involvement). The histological diagnosis of alcohol-related steatohepatitis was based on evidence of hepatocellular injury and inflammation.

### Statistics

Data are shown as median (range) or number (percentage) unless otherwise stated. A p-value of <0.05 was considered significant. Non-parametric analysis (GraphPad prism 5, San Diego, CA) utilising the Mann–Whitney U test or 1-way ANOVA (Kruskal-Wallis test) was applied where data were not distributed normally.

Logistic regression analysis was performed using SPSS 15.0 for Windows to study factors associated with fibrosis stage. Input variables were age, sex, presence of alcohol-related steatohepatitis and MELD score.

Log-rank test (Kaplan-Meier analysis) and Cox-regression model were used to identify predictors of clinical outcome. For the purpose of the Kaplan-Meier analysis alone the following parameters were divided into two groups based on their cohort medians: amount of alcohol consumed, hepatocyte p21 expression and MELD. In all other instances these parameters were treated as continuous variables. Only variables with a p-value of <0.10 on the univariate analysis were subjected to analysis multivariate analysis (Cox-regression).

## Results

### Patients

The clinical and histological characteristics of 42 patients within the spectrum of ALD (first cohort) are summarised in table 1. The median age was 51 years (28-69); 30 (71%) were men; 33 (43%) had ALD without histological evidence of active hepatitis; 32 (76%) were consuming alcohol to excess at the time of liver biopsy (median 86g/day (29-229)); the remainder had consumed alcohol to excess in the preceding 6 months. Patients were followed for a median 62 months (6-117) after liver biopsy. During follow-up, 22 of 32 (69%) of those who were consuming alcohol at the time of liver biopsy, continued to consume alcohol. During follow-up one patient developed hepatocellular carcinoma and nine died from liver-related causes. None underwent liver transplantation, either because of continued alcohol consumption or because of co-morbidity. Five other patients died of causes unlikely to be related to liver disease (trauma in 2 patients and 1 each with pneumonia, myocardial infarction and cerebrovascular disease).

**Table 1:** Clinical and histological characteristics of 42 patients with ALD (first cohort).

	**Median (range) or Number (percentage)**
**Histological features**	
Steatosis (0, 1, 2, 3)	13 (31%), 16 (38%), 9 (21%), 4 (10%)
Fibrosis (0, 1, 2, 3, 4)	1 (2%), 9 (22%), 8 (19%), 10 (24%), 14 (33%)
Alcohol related steatohepatitis - present / absent	24 (57%), 18 (43%)
**Biochemical parameters (normal range)**	
Albumin (30-51g/l)	35 (18-44)
Bilirubin (0-17µmol/l)	14 (3-291)
Alkaline phosphatase (30-135U/l)	150 (49-619)
Alanine transaminase (0-50U/l)	52 (27-176)
International Normalised Ratio	1.3 (0.9-1.5)
Platelets (150-400 x10^9^/l)	189 (65-408)
Creatinine (35-125µmol/l)	79 (40--119)
**Adverse liver-related outcome**	
Liver-related deaths	9 (21%)
Decompensation/Liver failure (+/- MOF)	6 (14%)
Variceal bleed	1 (2%)
ALD and sepsis	2 (5%)
Liver transplantation	0 (0%)
Hepatocellular carcinoma	1 (2%)

MOF: Multiorgan failure.

The clinical characteristics of 77 patients with histology confirmed alcohol-related cirrhosis (second cohort) are summarised in table 2; the median age was 50 years (26-80); 43 (56%) were men; all but one were consuming alcohol in excess at the time of liver biopsy (median 164g/day (57-600)). The median follow-up was 57 months (1-120) after liver biopsy. During follow-up, 46 of 76 (61%) of those who were consuming alcohol at the time of liver biopsy, continued to consume alcohol. During follow-up, 36 (47%) died of liver-related causes and two (3%) were considered for and underwent liver transplantation. A further five patients died of causes unlikely to be related to liver disease (1 each with pneumonia, metastatic cancer, congestive cardiac failure/ischaemic heart disease, chronic obstructive pulmonary disease and trauma).

**Table 2:** Clinical characteristics in 77 patients with ALD & cirrhosis (second cohort).

	**Median (range) or Number (percentage)**
**Biochemical parameters (normal range)**	
Albumin (30-51g/l)	26 (11-42)
Bilirubin (0-17µmol/l)	44 (7-627)
Alkaline phosphatase (30-135U/l)	307 (30-1760)
Alanine transaminase (0-50U/l)	34 (11-393)
International Normalised Ratio	1.4 (1.0-4.5)
Creatinine (35-125µmol/l)	73 (21-314)
**Adverse liver-related outcome**	
Liver-related deaths	36 (47%)
Decompensation (+/- MOF)	20 (26%)
Variceal bleed	13 (17%)
ALD and sepsis	3 (4%)
Liver transplantation	2 (3%)
Hepatocellular carcinoma	0 (0%)

MOF: Multiorgan failure.

### Distribution of hepatocyte cell cycle phase markers in the first cohort

Expression of hepatocyte Mcm-2, cyclin A, PH3 and p21 were negligible in normal liver tissue (<0.01% of hepatocytes in the background liver with colorectal cancer metastasis).

**Figure 1:**
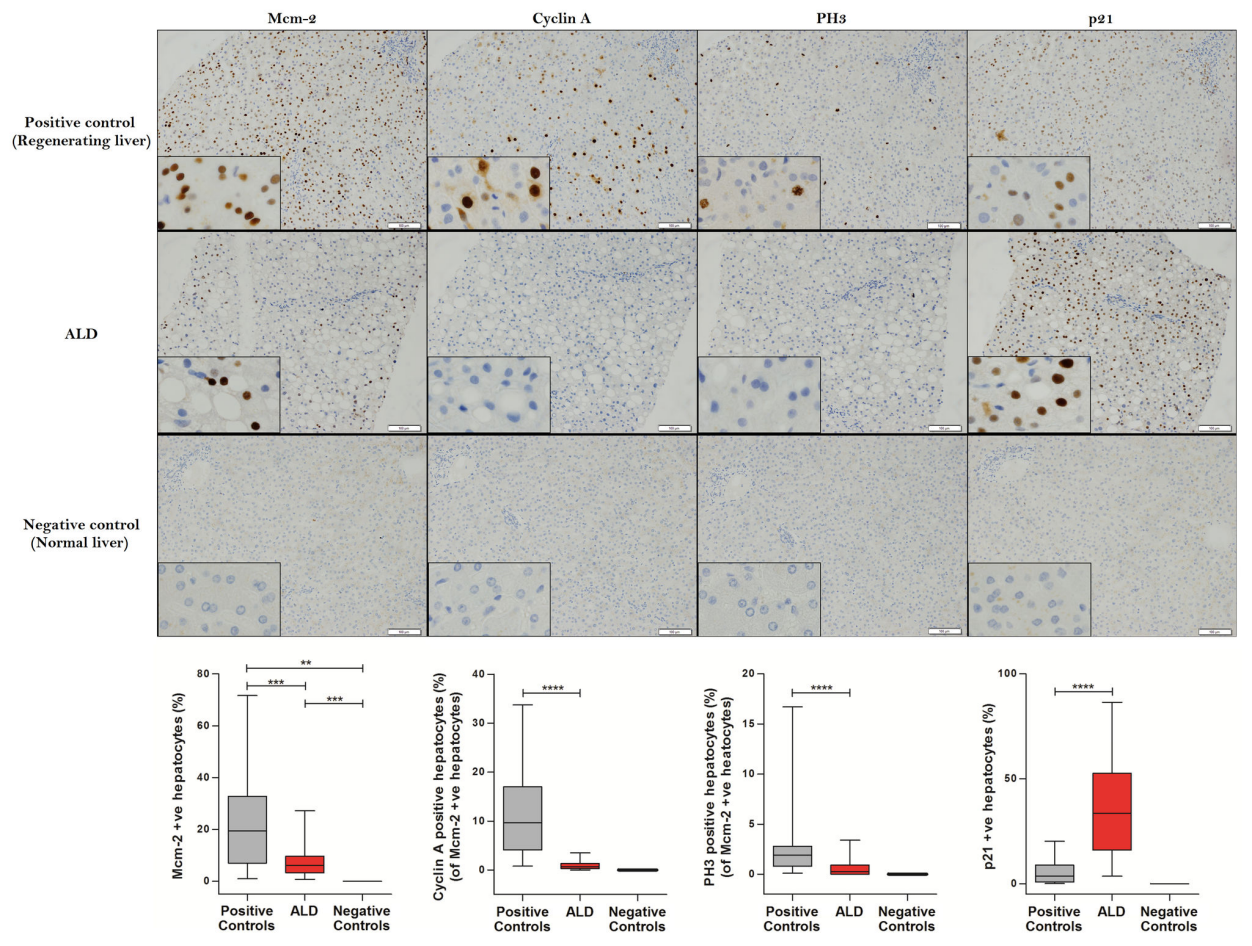
The distribution of cell cycle phase markers in the first cohort and controls. Examples of the immunohistochemical staining of Mcm-2, cyclin A, PH3 and p21 in regenerating liver (positive control tissue), liver from a representative patient with ALD and normal liver (negative control tissue). Hepatocyte Mcm-2 expression was higher in ALD and regenerating liver (positive control) compared to normal liver (negative control). Cyclin A and PH3 expression were lower in ALD compared to regenerating liver. In contrast hepatocyte p21 expression was higher in ALD compared to regenerating liver.

Mcm-2 expression was higher in hepatocytes in ALD and regenerative liver compared to normal liver, indicating cell cycle entry of hepatocytes in both conditions (p=0.0003 and p=0.001 respectively; Figure 1). However, hepatocyte Mcm-2 expression was lower in ALD compared to regenerating liver (7.3% vs. 22.7%; p=0.0009; Figure 1).

The proportion of hepatocytes that expressed cyclin A (S phase) in relation to the proportion of hepatocytes that were Mcm-2 positive was much lower in ALD compared to regenerating liver (0.9% and 11.3%, p<0.0001; Figure 1). The proportion of hepatocytes that expressed PH3 (M phase) in relation to the proportion of hepatocytes that were Mcm-2 positive was also lower in ALD compared to regenerating liver (0.5% and 2.9%, p<0.0001; Figure 1). Hepatocyte p21 (cell cycle inhibitor) expression was considerably higher in ALD compared to regenerating liver (35.6% vs. 5.6%, p<0.0001; Figure 1).

### Hepatocyte p21 expression and its associations - first and second cohorts

**Figure 2:**
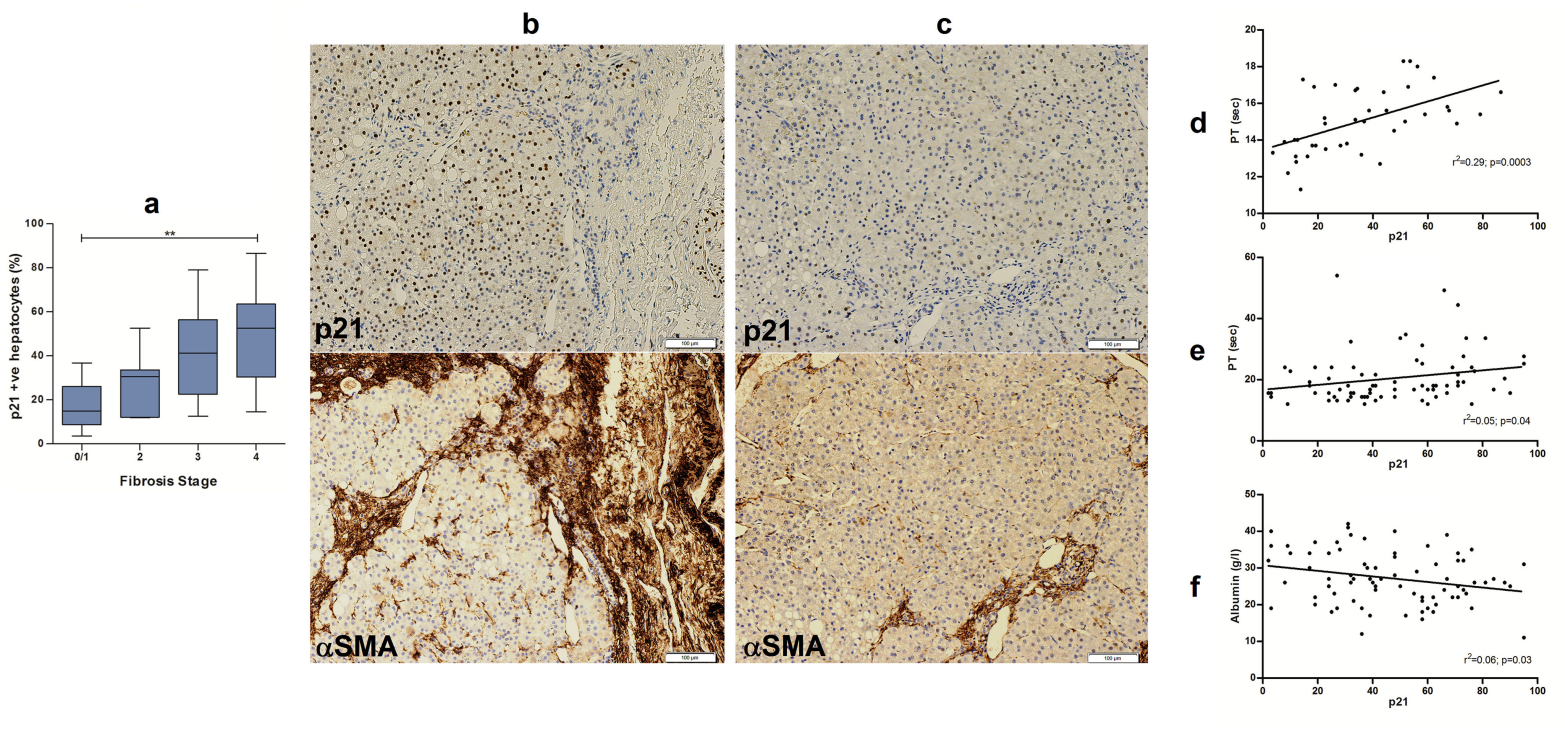
Hepatocyte p21 expression in relation to fibrosis stage and laboratory indices. Hepatocyte p21 expression demonstrated an association with fibrosis stage [2a] in the first cohort; the proportion of hepatocytes that expressed p21 increased with increasing fibrosis stage. Areas of increased α-SMA expression (a marker of activated hepatic stellate cells) were associated with higher hepatocyte p21 expression (2b) than those areas with less α-SMA expression, which were associated with lower hepatocyte p21 expression (2c) even within the same tissue. Expression of hepatocyte p21 correlated positively with prothrombin time in both cohorts (Figure 2d & 2e, respectively) and correlated negatively with serum albumin in the second cohort (Figure 2f).

**Figure 3:**
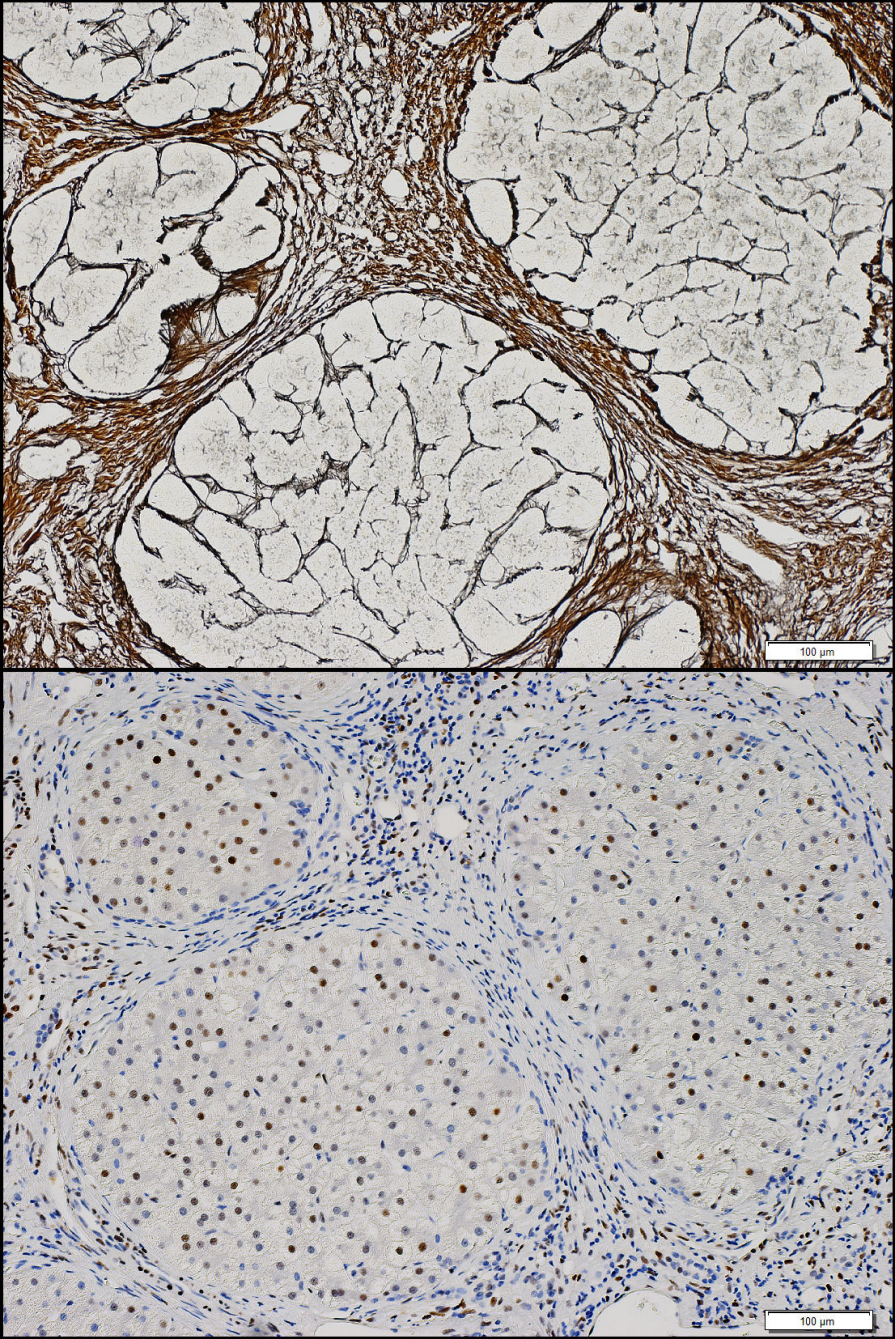
Differential hepatocyte p21 expression in the second cohort. Figure 3 shows fibrosis using reticulin staining (above) and hepatocyte p21 expression (below) in a patient with ALD cirrhosis in second cohort.

In the first cohort, there was a positive correlation between hepatocyte p21 expression and fibrosis stage (p=0.002; Figure 2), which remained significant (p=0.005) after multivariate analysis when controlled for age, sex, presence of alcohol-related steatohepatitis and MELD score. A similar analysis could not be undertaken in the second cohort as it only included patients with established cirrhosis (Figure 3).

Areas of liver with increased hepatic stellate cell activation were associated with higher hepatocyte p21 expression; in contrast, areas of liver with fewer activated hepatic stellate cells were associated with lower hepatocyte p21 expression, even within the same tissue (Figure 2).

Expression of p21 was diffuse and not restricted to periportal or perivenular zones. Hepatocyte p21 expression was not associated in either cohort with the steatosis grade (p=0.14 and p=0.36, respectively) or with the presence of alcohol-related steatohepatitis (p=0.07 and p=0.20, respectively).

Hepatocyte p21 expression demonstrated no correlation with either MELD score (p=0.11 and p=0.36) or the amount of alcohol consumed at the time of liver biopsy (p=0.20 and p=0.94) in the first and second cohorts, respectively. In addition, in the first cohort, there was no difference in hepatocyte p21 expression between those who were consuming and those not consuming alcohol at the time of liver biopsy (p=0.07). A similar analysis was not carried out in the second cohort, as only one patient was not consuming alcohol at the time of liver biopsy.

In the first cohort, hepatocyte p21 expression correlated positively with prothrombin time (p=0.0003; Figure 2) but showed no association with serum albumin level (p=0.13). In the second cohort, however, hepatocyte p21 expression demonstrated a positive correlation with prothrombin time (p=0.04; Figure 2) and an inverse correlation with serum albumin (p=0.03; Figure 2). Hepatocyte p21 expression was not associated with serum total bilirubin in either cohort (p=0.46 and p=0.62, respectively).

### Hepatocyte p21 expression and outcome - first and second cohorts

**Figure 4:**
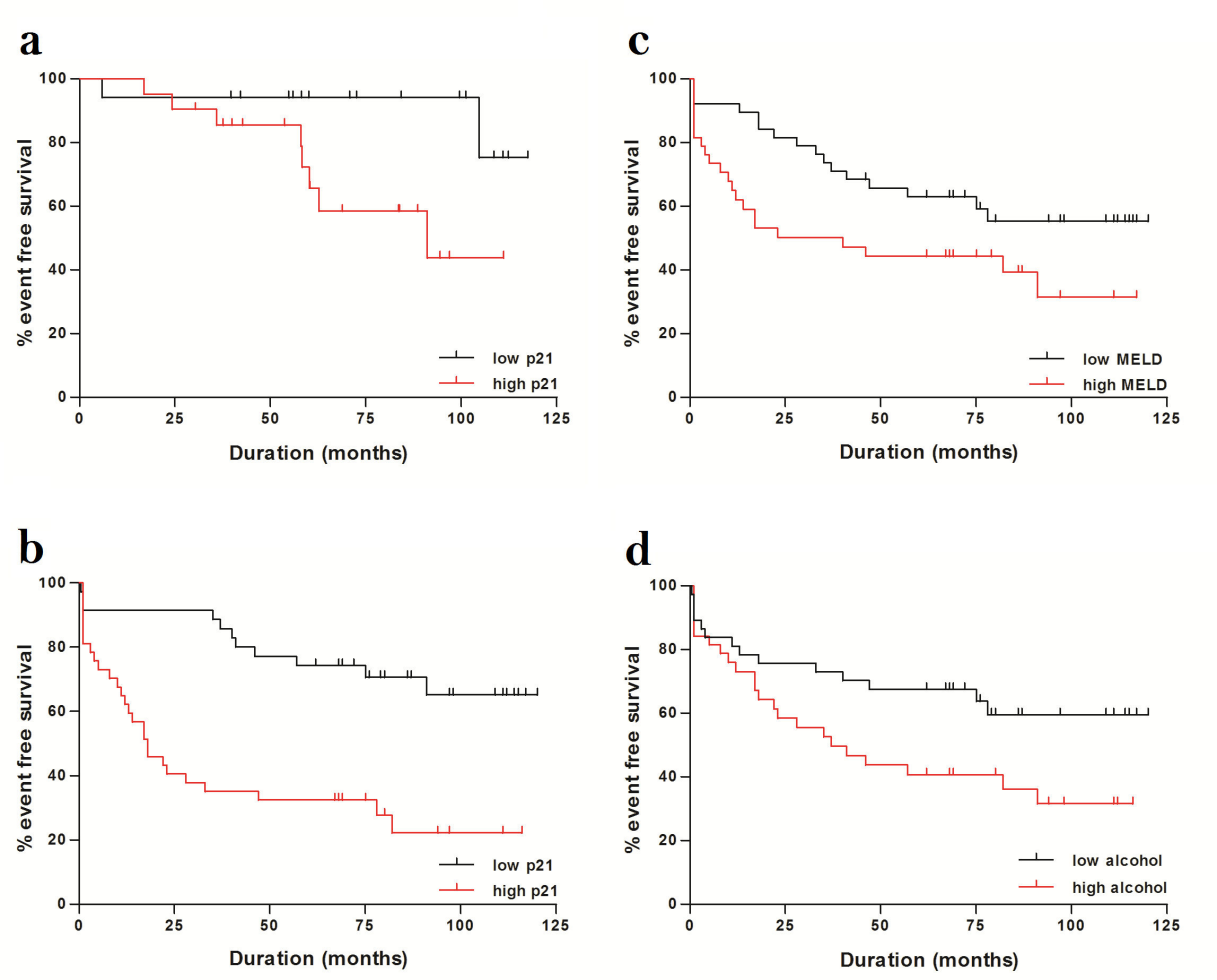
Hepatocyte p21 expression, MELD score and alcohol consumption at the time of liver biopsy in relation to an adverse liver-related outcome. Kaplan-Meier analysis in 42 patients in the first cohort, divided according to hepatocyte p21 expression above the median [red line] or below the median [black line]) from the time of liver biopsy to outcome or censor point by log-rank test (p=0.03) [4a]. Kaplan-Meier analysis of 77 patients in the second cohort from the time of liver biopsy to outcome or censor point by log-rank test - divided according to p21 expression above the median [red line] or below the median [black line] (p<0.0001) [4b]; MELD score above the median [red line] or MELD score below the median [black line] (p=0.03) [4c]; alcohol consumption above the median [red line] or alcohol consumption below the median [black line] (p=0.03) [4d].

Analysis with the Log-rank test (univariate analysis) in the first cohort revealed that patients with higher hepatocyte p21 expression (above the median) were more likely to develop an adverse liver-related outcome than those with lower p21 expression (p=0.03; Figure 4a). Age (p=0.91), sex (p=0.65), fibrosis stage (p=0.83), alcohol-related steatohepatitis (p=0.82), MELD score (p=0.52), the quantity of alcohol consumed at the time of liver biopsy (p=0.22) and continued alcohol consumption during follow-up (p=0.11) were not related to an adverse liver-related outcome. Multivariate analysis was not performed in the first cohort as only p21 expression demonstrated an association with liver-related outcome (with p-value <0.10) on univariate analysis.

**Figure 5:**
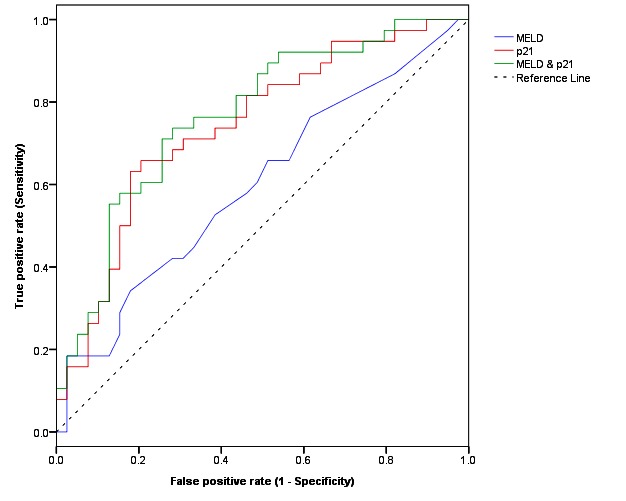
ROC curves comparing hepatocyte p21 expression alone, MELD score alone and hepatocyte p21 expression plus MELD score in predicting an adverse liver related outcome in patients with alcohol-related cirrhosis. The area under the receiver operating characteristic (ROC) curves for hepatocyte p21 expression alone (0.74), MELD score alone (0.59) and hepatocyte p21 plus MELD score (0.76) are shown

Analysis with the Log-rank test (univariate analysis) in the second cohort revealed that patients with higher hepatocyte p21 expression (p < 0.0001; Figure 4b), higher MELD score (p=0.03; Figure 4c) and excess alcohol consumption at the time of biopsy (p=0.03; Figure 4d) were each associated with an adverse liver-related outcome (table 3). As with the first cohort, age (p=0.43), sex (p=0.27), alcohol-related steatohepatitis (p=0.51) and alcohol consumption during follow-up (p=0.55) were not related to outcome. On Cox regression analysis (multivariate analysis) both MELD score (HR=1.05; 95% CIHR 1.01-1.10; p=0.008) and hepatocyte p21 expression (HR=24.56; 95% CIHR 5.10-118.35; p<0.001) but not the quantity of alcohol consumed at the time of biopsy (HR=0.99; 95% CIHR 0.99-1.00; p=0.07), were associated independently with an adverse liver-related outcome (table 3). However, hepatocyte p21 expression was associated more closely with an adverse liver-related outcome (AUROC 0.74; 95% CI 0.63-0.85; p=0.0002; Figure 5) than MELD score (AUROC 0.59; 95% CI 0.47-0.72; p=0.13; Figure 5). Combining MELD score with hepatocyte p21 expression made no improvement to the predictive value of hepatocyte p21 expression alone (AUROC of MELD score plus p21 0.76 versus AUROC of hepatocyte p21 alone 0.74; p=0.70). With a cut-off at 25%, 33% and 50%, hepatocyte p21 expression had 95%, 84% and 66% sensitivity and 33%, 49% and 77% specificity, respectively, in predicting an adverse liver-related outcome.

**Table 3:** Univariate and multivariate analysis of factors that influence an adverse liver-related outcome in the second cohort.

	**Univariate analysis**		**Multivariate analysis**	
	**HR (95% CIHR)**	**p-value**	**HR (95% CIHR)**	**p-value**
Age	0.77 (0.40-1.48)	0.43		
Sex	0.69 (0.36-1.33)	0.27		
Alcohol consumption at biopsy	2.02 (1.05-3.92)	0.03	0.99 (0.99-1.00)	0.076
Alcoholic steatohepatitis	1.36 (0.47-3.97)	0.51		
MELD score	2.03 (1.05-3.93)	0.03	1.05 (1.01-1.10)	0.006
p21	3.97 (2.03-7.77)	< 0.0001	24.56 (5.10-118.35)	< 0.001
Alcohol consumption during follow-up	1.21 (0.62-2.36)	0.55		

In the second cohort, the time to an adverse liver-related outcome was much shorter in patients with hepatocyte p21 expression above the median compared to patients with lower hepatocyte p21 expression (12 months (1-82) versus 40 months (1-90); p=0.04).

## Discussion

In this study, the distribution of hepatocyte cell cycle phase markers and hepatocyte p21 expression were examined in the first cohort, which was selected carefully from a larger series such that alcohol was the only known risk factor for liver disease, long-term follow-up was assured, liver sections were of adequate size and the patients covered a broad spectrum of liver histology. This revealed impaired hepatocyte cell cycle progression beyond G_1_/S phase and increased hepatocyte p21 expression. The association between hepatocyte p21 expression and a subsequent adverse liver-related outcome prompted further investigation. Thus, a further well-defined group of patients that had undergone liver biopsy and long term follow-up were recruited to study the association between hepatocyte p21 expression and clinical outcome. Since hepatocyte p21 expression was higher in advanced fibrosis and an adverse liver-related outcome is more common in patients with advanced fibrosis, the second cohort was selected such that all patients had ALD cirrhosis. The strong relation between increased hepatocyte p21 expression and an adverse liver-related outcome was confirmed in this second cohort. These data are consistent with previous data in patients with non-alcohol-related fatty liver disease which revealed a close link between hepatocyte p21 expression, fibrosis stage and increased liver related morbidity and mortality [12].

Oxidative stress, such as ethanol-induced oxidative stress, causes double-strand DNA breaks [13], which in turn leads to cellular senescence and permanent cell cycle arrest [8]. Such senescent cells display DNA content typical of the G_1_ phase of cell cycle [9]. The presence of Mcm-2, which is present throughout the cell cycle, in both regenerating liver and ALD shows the replicative response of hepatocyte to acute ischaemia and alcohol-related insult, respectively. However, the relative reduction in Cyclin A expression (marker of S phase) and PH3 expression (marker of M phase) in ALD compared to regenerating liver indicates impaired cell cycle progression beyond the G_1_/S phase in ALD. This is corroborated by a marked increase in hepatocyte p21 expression in ALD compared to regenerating liver, since p21 is a universal cell cycle inhibitor which exerts its effect at the G_1_/S phase transition [14]. It was notable that hepatocyte p21 expression in ALD exceeded Mcm-2 expression substantially; this disparity is not explained readily. This may be due to depletion of Mcm proteins in hepatocytes in permanent cell cycle arrest as described previously [15] to prevent completion of mitosis or direct induction of p21, perhaps due to cellular stress, in hepatocytes that have not entered the cell cycle.

There was progressive increase in hepatocyte p21 expression with increasing fibrosis stage in ALD. This observation, together with the increase in hepatocyte nuclear area in ALD cirrhosis described previously [7] and previous data linking both increased hepatocyte p21 expression and increased hepatocyte nuclear area with fibrosis stage in non-alcohol-related fatty liver disease [12], strengthens the notion that senescent hepatocytes accumulate with progressive fibrosis. It is unclear whether senescent hepatocytes drive fibrosis, perhaps by activating hepatic stellate cells, or if hepatocyte senescence and fibrosis are both consequences of the same alcohol-related insult. The geographic association between hepatocyte p21 expression and hepatic stellate cell activation (shown by α-SMA expression) demonstrated in this study supports the notion that hepatocyte senescence, activation of hepatic stellate cells and fibrosis are linked. A recent study shone light on the causal relation between hepatocyte senescence and hepatic stellate cell activation by demonstrating reduced collagen deposition, reduced α-SMA, a marker of hepatic stellate cell activation, and reduced hepatic p21 expression, the downstream target of p53, in p53-deficient mice after 8 weeks of MCD diet [16].

Hepatocyte proliferation is critical for liver regeneration [17]. Accumulation of senescent hepatocytes, which are insensitive to mitotic stimuli, may impair the reserve for liver regeneration. In addition, the association between increased hepatic nuclear area and hepatic dysfunction [7,12] suggests that senescent hepatocytes may not function as normal mature hepatocytes. In the present study, there was also an association between senescence, measured as increased hepatocyte p21 expression and impaired liver synthetic function (increasing prothrombin time and decreasing serum albumin level). Thus, accumulation of senescent hepatocytes may contribute to loss of functional hepatic mass and with sufficient accumulation of such cells leading eventually to liver decompensation and liver related death, accounting for the strong link between hepatocyte p21 expression and an adverse liver-related outcome. Previous studies in cells other than hepatocytes have shown loss of function specific to that cell type during senescence [18]. If this proves true for hepatocytes in ALD, then that might explain, at least in part, the clinical and biochemical dysfunction with progressive liver disease - since up to 87% of hepatocytes in ALD in this series expressed p21 and may have impaired function. The absence of an association between hepatocyte p21 expression and serum bilirubin, an important maker of liver dysfunction in general and in ALD in particular, suggests that an elevated bilirubin is not a surrogate marker of senescence and may perhaps reflect superimposed bacterial sepsis, which is common in ALD.

The relation between hepatocyte p21 expression and outcome is noteworthy. Indeed in this series with ALD, the relation between hepatocyte p21 expression and outcome was stronger than that for MELD score, fibrosis stage, alcohol-related steatohepatitis or continued alcohol consumption. Hepatocyte p21 expression can be assessed readily by immunohistochemistry (much more readily than nuclear area) and has the potential to be useful for assessing prognosis in day-to-day clinical practice, stratification in future clinical trials in ALD and even when liver transplantation is being considered and the prognosis remains uncertain.

The first cohort comprised a consecutive series of patients with all stages of fibrosis and all manifestations of alcohol-related steatohepatitis. Although patients were selected for single aetiology, tissue quality and extended follow-up, in every other respect, this cohort reflects ‘real world everyday practice’. The second cohort included only patients with cirrhosis but with all grades of alcohol-related steatohepatitis. Both cohorts were selected carefully only to include those with biopsy-proven ALD, those with adequate tissue for meaningful analysis, with adequate long-term follow-up and all necessary data available including information on alcohol consumption during follow-up.

Information on the quantity of alcohol consumed during follow-up was self-reported and may be less accurate and this remains a limitation of the study. A further limitation was that information regarding nutritional status, which may have influenced the clinical outcome, was not available in a sufficient number of patients for analysis.

In conclusion, this study demonstrated that impaired cell cycle progression in hepatocytes, hepatocyte senescence and progressive fibrosis in ALD were inter-related. Further, there was a strong relation between the proportion of senescent hepatocytes and an adverse liver-related outcome. Similar findings were demonstrated in non-alcohol-related fatty liver disease [12]. It is plausible that cellular senescence is a common response of the hepatocyte to cellular stress and injury and not limited to a particular aetiology.
